# Metabolic syndrome and liver-related events: a systematic review and meta-analysis

**DOI:** 10.1186/s12902-019-0366-3

**Published:** 2019-04-25

**Authors:** Huina Ren, Junna Wang, Yue Gao, Fuwei Yang, Wenxiang Huang

**Affiliations:** Department of Infectious Diseases, The First Affiliated Hospital of Chongqing Medical University, Chongqing, 400016 China

**Keywords:** Metabolic syndrome, Diabetes mellitus, Insulin resistance, Metabolic abnormalities, Hepatocellular carcinoma, Cirrhosis, Liver-related events, Meta-analysis

## Abstract

**Background:**

Previous studies have suggested that metabolic syndrome (MetS) and its component conditions are linked to the development of many benign or malignant diseases. Some studies have described relationships among metabolic syndrome or diabetes and liver cancer, but not many articles described the relationships between MetS and cirrhosis, acute hepatic failure, end-stage liver disease, and even death. However, liver cancers, cirrhosis, acute hepatic failure, end-stage liver disease, and liver-related mortality—collectively described as liver-related events (LREs)—may have different relationships with MetS. We undertook this meta-analysis to examine the association between MetS and LREs, and to determine whether geographic region or hepatitis B virus (HBV) positivity might influence the association.

**Methods:**

Relevant studies were identified from PubMed, EMBASE, and the Cochrane database. Two reviewers independently searched records from January 1980 to December 2017. The search terms included ‘metabolic syndrome’, ‘diabetes mellitus’, ‘insulin resistance syndrome’, and ‘metabolic abnormalities’, combined with ‘cirrhosis’, ‘hepatic fibrosis ’, ‘hepatocellular carcinoma’, ‘complication’, ‘LRE’, ‘HCC’, ‘liver-related events’, and ‘liver cancer’. No language restriction was applied to the search. We chose the studies reporting an association between MetS and LREs. We used Begg’s and Egger’s tests and visually examined a funnel plot to assess publication bias. All analyses were conducted in Stata 14.0 software.

**Results:**

There were 19 studies (18 cohort and 1 case-control) included in the analysis, with a total of 1,561,457 participants. The subjects’ ages ranged from 18 to 84 years. The combined analysis showed an overall 86% increase risk of LREs in cases with MetS (RR: 1.86,95% CI: 1.56–2.23). The funnel plot was asymmetrical, and the Egger’s test *p* values showed a publication bias in this meta analysis. However, through the trim and fill method, we obtained a new RR value for LREs with MetS of 1.49 (95% CI: 1.40–1.58, *p* = 0.000). There was no obvious difference with the two answers, so we concluded that the results were robust. For hepatitis B positive patients, the RR for MetS and LREs was 2.15 (95% CI:1.02–4.53, *p* = 0.038), but for the hepatitis B negative patients, the RR was 1.85 (95% CI:1.53–2.24, *p* = 0.000). And for non-Asians, the RR for MetS and LREs was 2.21 (95% CI: 1.66–2.69, *p* = 0.000), while for Asians, the RR was 1.73 (95% CI: 1.35–2.22, *p* = 0.000).

**Conclusions:**

This meta-analysis showed that MetS is associated with a moderately increased risk of LREs prevalence. Patients with MetS together with hepatitis B are more likely to develop hepatic events. For non-Asians, MetS is more likely to increase the incidence of LREs.

## Background

Because of a significant increase in the incidence and mortality of hepatocellular carcinoma (HCC), this cancer has become one of the most common malignancies and a major cause of death worldwide. In recent years, chronic liver disease has become a major cause of death in the United States, causing a large number of deaths every year, according to national life statistics. All the cirrhotic complications, HCC, and/or liver-related mortality were called LREs. And liver-related death is defined as death related to hepatic events [1]. Viral hepatitis and excessive drinking have been identified as the major risk factors for LREs, but risk factors for approximately 5 to 30% of HCC cases remain to be identified [2, 3]. Metabolic syndrome (MetS) patients have a high risk of cardiovascular disease. In addition, there is increasing evidence that patients with chronic liver disease are at risk of a higher rate of diagnosis of MetS or diabetes mellitus [4]. Recent studies have shown that MetS might have additional associations with liver diseas*e.* Liver cancer is the most severe liver disease. Although MetS is known to promote liver cancer, there is little evidence of whether MetS is associated with cirrhosis, liver failure, liver fibrosis, and death from liver causes.

MetS has become a major public health focus worldwide, and it is related to the occurrence of obesity and the diabetes pandemic. MetS components are a series of risk factors for cardiovascular diseases and it has become an increasingly severe problem globally [5]. The risk factors comprising MetS include obesity, abnormal blood sugar, Increased blood pressure, elevated triglyceride levels, decreased high-density lipoprotein cholesterol levels [6]. Several studies have described the relationship between MetS and diabetes and liver cancer [5], but few have investigated the LREs. The article intended to address this knowledge gap. Recent cohort studies have attempted to further understand the temporal relationship between these factors and to validate previous findings. However, there are few data on the association of LREs and MetS factors, which include obesity, diabetes and MetS.

In this study, we conducted a systematic review and meta-analysis to assess all available evidence to identify an association between MetS and LREs. We additionally analyzed related factors.

## Methods

### Search strategy

We used PubMed, EMBASE, and the Cochrane database for literature retrieval. In December 2017, the following search terms were used without language restrictions: MetS, diabetes mellitus, insulin resistance, metabolic abnormalities, cirrhosis, hepatic fibrosis, hepatocellular carcinoma, complication, LRE, HCC, liver-related event, and liver cancer All references cited in these studies were also reviewed to identify other published articles not indexed in the databases. The systematic review process followed established quality standards for reporting of meta-analyses.

### Study eligibility

The inclusion criteria for studies in the meta-analysis were as follows: either case-control or cohort design; inclusion of subjects over 18 years old; assessment of the effects of MetS on the risk of liver events; and reporting of relative risk (RR) estimates for LREs in subjects with MetS. Only complete papers and published studies in the medical literature were included. Data from summaries, reviews, editorials, case reports, and letters were excluded. Studies reporting no risk ratio (HR) and 95% confidence intervals (95% CI), and those with participants with incomplete data were also excluded.

### Data extraction

The following data were collected from each study: first author’s surname, type of article, year and country of publication, distribution of age and sex, number and characteristics of the participator, definitions of MetS, risk estimates with corresponding confidence intervals (CIs), and all other information. Quality assessment for cohort studies in this meta-analysis was assessed with the Newcastle Ottawa scale (NOS), as recommended by the Cochrane Non-Randomized Studies Methods Working Group. The scale allocates a number of stars ranging from one to nine.

### Statistical analysis

We used both fixed- and random-effects models to calculate the pooled RR and 95% CI. We performed an analysis to identify the association between MetS and LREs, and also to analyze any influence of geographic region or HBV positivity. Heterogeneity was assessed with the *I*^2^ statistic and *p* value. Funnel plots and Egger and Begg’s and Mazumdar’s tests were used to assess publication bias. A *p* value < 0.05 was considered statistically significant [7, 8]. The trim and fill method aims to identify and correct the funnel asymmetry caused by publication bias, it can remove a small sample study that causes asymmetry in the funnel plot, and then estimate the center value of the funnel plot with the symmetrical part after trimming. All statistical analysis was performed in Stata 14.0 software.

## Results

**Fig. 1:**
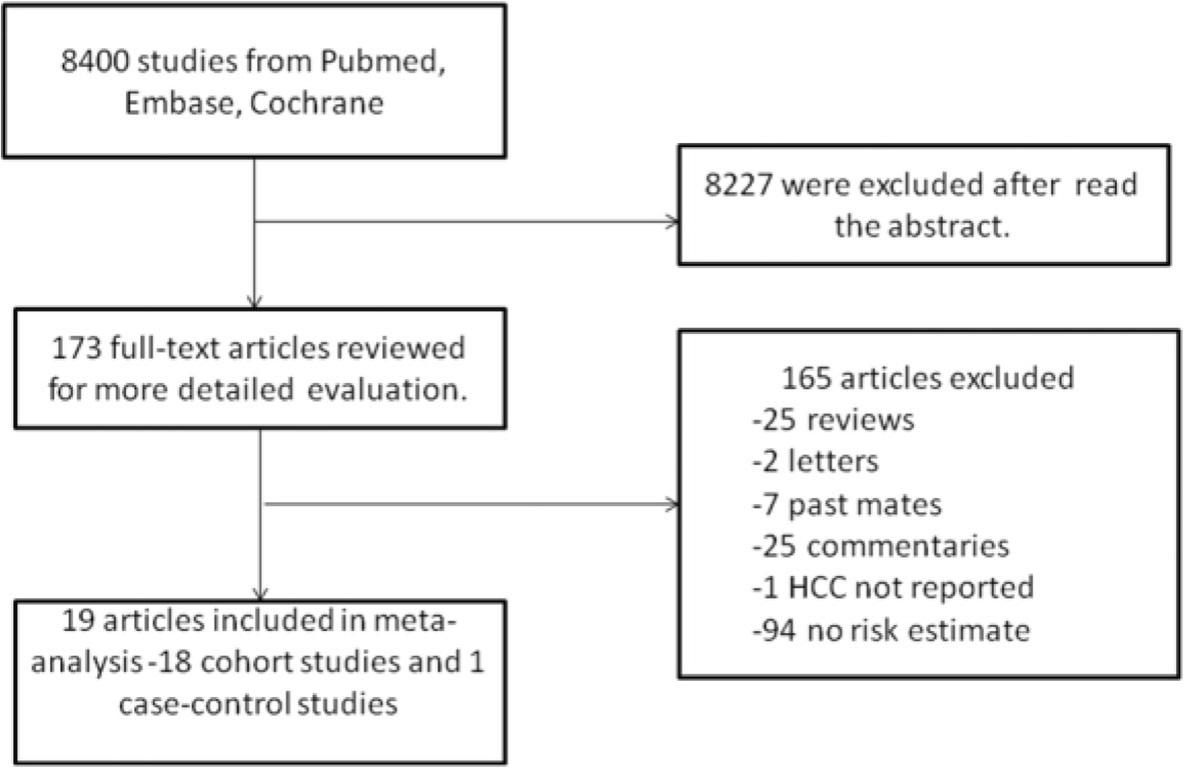
Flow chart of study selection in this systematic review

Figure 1 details our search steps. A total of 1,561,457 individuals were included in the study. Overall, we identified 8400 studies from different databases, including 4559 studies from PubMed, 173 studies from the Cochrane database, and 3668 studies from EMABASE. After reading the abstract, we initially considered 173 publications to be relevant. Among them, we excluded 25 comments, 2 letters, 7 meta-analysis, 25 comments, 1 unreported HCC, and 94 lacking risk estimates. A total of 19 studies (18 cohort studies and one case-control study) were included in the final analysis [1, 6, 9–25].

### Association between metabolic syndrome and liver-related events

**Fig. 2:**
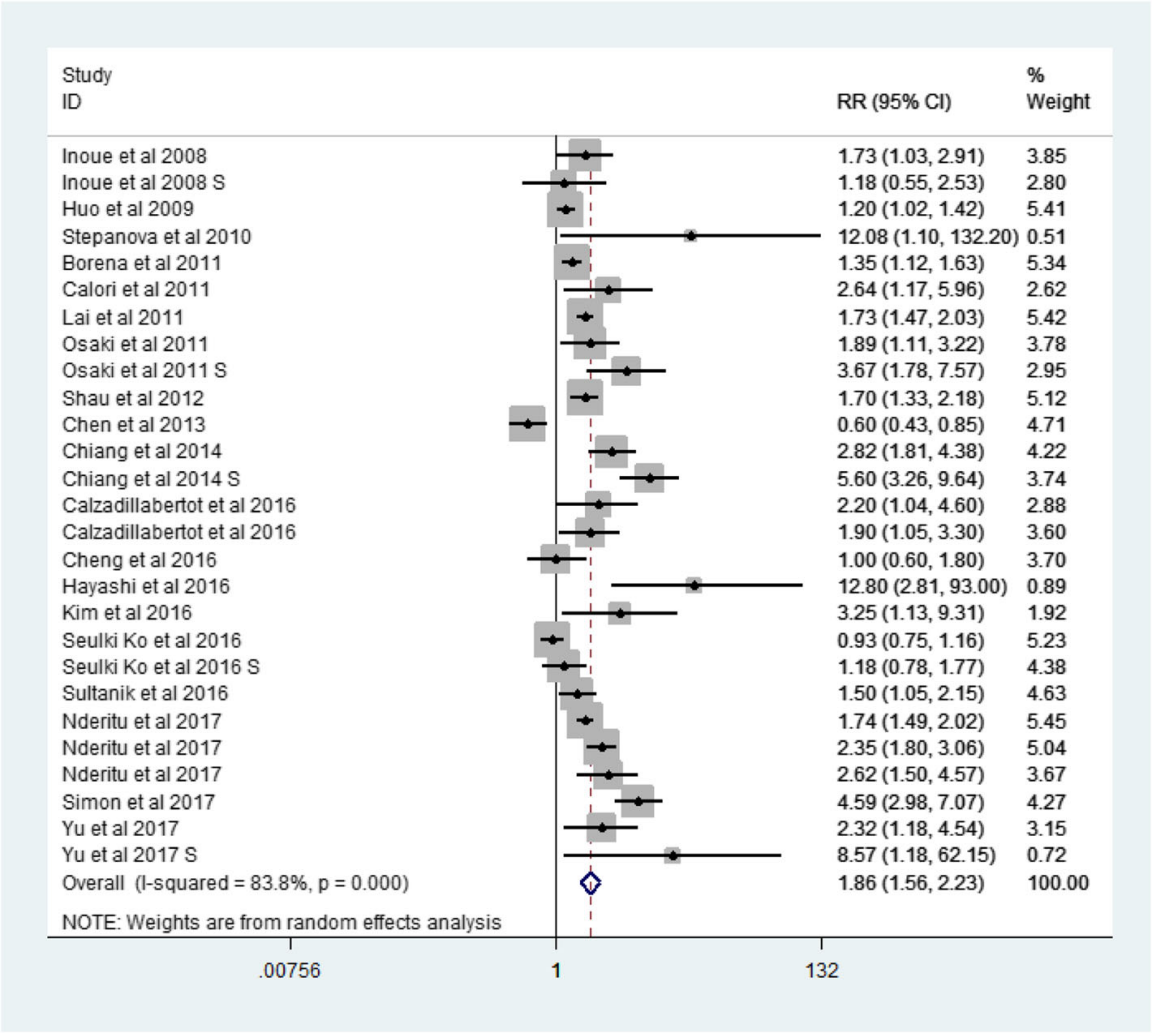
Forest plot: Meta-analysis of the association between metabolic syndrome and liver-related events

A total of 19 articles were included in this meta analysis. Among the 18 cohort studies, there were 1,546,392 participants, of which 7373 had liver-related events (LREs) (Table 1). The one case-control study was published in 2010 as part of the Third National Health and Nutrition Examination Survey, and it included 2061 liver disease cases and 13,004 controls. The age of participants ranged from 18 to 84 years old. The pooled RR (among 1,561,457 participants from 19 studies) for LREs among subjects with MetS was 1.86 (95% CI, 1.56–2.23) (Fig. 2).

**Table 1:** Main characteristic of 19 eligible studies in this meta-analysis

Study authors,	Country	Type of study	Study population	Age(years)	Male:Female	N-O-Scale	RR,95%CI, and p	Follow-up period	Definition of Metabolic risk factors	Adjustment
Inoue et al.,2008 [9]	Japan	Cohort (prospective cohort)	27,724 total Japanese men and women (114 HCC cases)	Men:56.5 years, Women:55.5 years	9548:18,176	8	M-1.73,1.03–2.91,P=NA F-1.18,0.55–2.51,P=NA	9–11 years	AHA	Age, sex, study area, smoking status, weekly ethanol intake
Huo et al.,2009 [10]	China	Cohort (prospective cohort)	1713 HCC cases(907 HCC-related died)	65.4 years	1313:400	9	Total-1.2,1.02–1.42,P = 0.03	18 ± 16 months	WHO	Age, sex
Stepanova et al.,2010 [11]	Europe and America	Case-control (retrospective cohort)	2061 liver disease (331 died) 13,004 controls(177 died)	Case:41.1 years, Controls:44.4 years	Case:192:139 Controls:5943:7061	8	Total-12.08,1.1–132.2,*P* = 0.042	12–18 years	WHO	Age, sex, smoking status
Borena et al.,2011 [12]	Europe	Cohort (prospective cohort)	578,700 people from Norway, Austria and Sweden(266 HCC cases)	Men:43.9 years, Women:44.1 years	289,866:288,834	8	Total-1.35,1.12–1.63,P=NA	Men for 12.8 years, Women for 11.3 years	WHO	Age, sex, smoking status
Calori et al.,2011 [13]	Italy	Cohort (prospective cohort)	2011 total Italians (34 liver-specific died)	57 years	885:1126	8	Total-2.643,1.172–5.957, *P* < 0.0191	15 years	WHO	Age, sex,smoking status, alcohol intake
Lai et al.,2011 [14]	China	Cohort (retrospective cohort)	19,349 total Chinese (224 HCC cases)	55.5 years	10,792:8557	7	Total-1.73,1.47–2.03, P=NA	3–8 years	ICD	Age, sex
Osaki et al.,2011 [15]	Japan	Cohort (prospective cohort	23,625 Japanese men and women(129 HCC cases)	58.6 years	8239:15,386	7	M-1.89,1.11–3.22, F-3.67,1.78–7.57, P=NA	9.1 years	NCEP-ATP III	Age, sex,smoking status, heavy drinking
Shau et al.,2012 [16]	China	Cohort (prospective cohort	931 HCC cases who received surgical resection(321 liver-specific died)	57.7 years	679:252	9	Total-1.7,1.33–2.18, *P* < 0.001	5–6 years	NCEP-ATP III	Age,sex,tumor stage
Chen et al.,2013 [6]	China	Cohort (prospective cohort	56,231 total Chinese men and women(262 HCC cases)	60.9 years	17,440:38,896	6	Total-0.6,0.43–0.85, *P* = 0.004	5–7 years	AHA	Age, sex, weight, liver function
Chiang et al.,2014 [17]	China	Cohort (prospective cohort	50,080 Chinese men and women (235 HCC-related deaths)	M:54.2 years, F:53.7 years	23,484:26,596	9	M-2.82,1.81–4.38, *P* < 0.0001 F-5.60,3.26–9.64, *P* < 0.0001	10 years	WHO	Age,sex,smoking status,alcohol intake
Calzadillabertot et al.,2016 [18]	Cuba	Cohort (prospective cohort	250 compensated HCV-related cirrhosis(28 death and 55 decompensated)	60 years	96:154	8	Died-2.2,1.04–4.6, P = 0.04 Decompensated- 1.9,1.05–3.3, P = 0.03	22–80 months	WHO	Age,sex,alcohol intake
Cheng et al.,2016 [1]	China	Cohort (prospective cohort	1466 CHB patients(93 hepatic events)	46 years	939:527	8	Total-1.0,0.6–1.8, *P* = 0.939	88 ± 20 months	NCEP-ATP III	Age, sex, weight, weekly ethanol intake, liver function
Hayashi et al.,2016 [19]	Japan	Cohort (prospective cohort	474 Japanese non-cirrhotic patients with chronic hepatitis (21HCC cases)	58 years	230:244	9	Total-12.8,2.81–93.0, *P* = 0.0006	8 years	HOMA	Age,sex
Kim et al.,2016 [20]	Korea	Cohort (prospective cohort	1696 chronic HBV infected patients (24 HCC cases)	50 years	964:732	9	Total-3.25,1.13–9.31, *P* = 0.028	1.0–10.5 years	NCEP-ATP III	Age,sex
Seulki Ko et al.,2016 [21]	Korea	Cohort (prospective cohort	99,565 Koreas men and women(588 HCC cases)	Above 20 years	61,758:37,807	8	M-0.93,0.75–1.16, P=NA F-1.18,0.78–1.77, P=NA	10.4 years	WHO	Age,sex,smoking status,alcohol intake
Sultanik et al.,2016 [22]	France	Cohort (prospective cohort	341 HCV patients with cirrhosis(136 HCC cases,ESLD cases,HCC and ESLD cases)	56 years	225:116	9	Total-1.5,1.05–2.15, P = 0.03	8.75 years	WHO	Age,sex,alcohol intake
Nderitu et al., 2017 [23]	Sweden	Cohort (prospective cohort	509,436 participants (2775 cirrhosis cases,766HCC cases,158 cirrhosis and HCC cases)	44 years	272,167:237,269	9	Cirrhosis-1.74,1.49–2.02, HCC-2.35,1.80–3.06, LREs-2.62,1.50–4.57, P=NA	13.6 years	WHO	Age,sex
Simon et al.,2017 [24]	USA	Cohort (prospective cohort	171,110 Americans men and women (112 HCC cases)	64.1 years	50,284:120,826	9	Total-4.59,2.98–7.07, P < 0.0001	32 years	National Diabetes Date Group	Age,sex,race,smoking status,alcohol intake
Yu et al.,2017 [25]	China	Cohort (prospective cohort	1690HBV carriers (158HCC cases,126 liver-related died)	48.4 years	All men	7	HCC-2.32,1.18–4.54, P=NA LREs-8.57,1.18–62.15, P=NA	19 years	Asian and Chinese Criteria	Age,smoking status,alcohol intake

*MetS* Metabolic syndrome, *HCC* hepatocellular carcinoma, *RR* relative risk, N-O-Scale Newcastle-Ottawa quality assessment scale, *LREs* Liver-related events, ICD – International Classification of Disease, *HOMA* Homeostasis Model Assessment, *WHO* World Health Organization,*NCEP-ATP III* National Cholesterol Education Program-Adult Treatment Panel III, *AHA* American Heart Association, *M* male,*F* female, *NA* not applicable

**Fig. 3:**
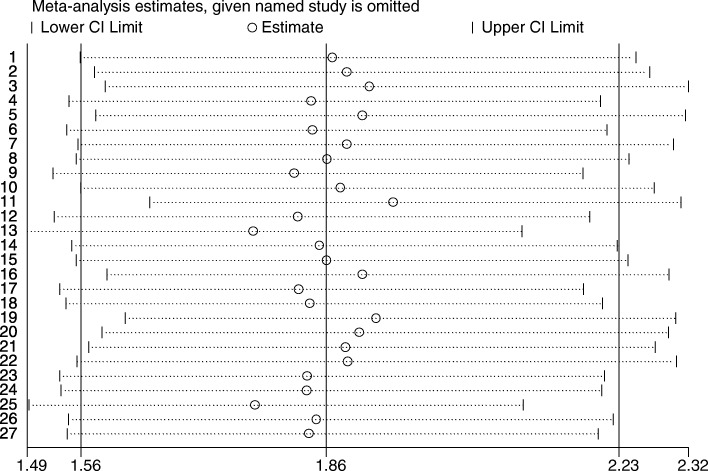
Meta-analysis random-effects estimates

Because of the heterogeneity of the 19 studies (*p* value for heterogeneity = 0.000, I2 = 83.8%) (Fig. 2), we used the random-effects model to calculate the combined RR (Fig. 3). On the basis of the random-effects model, no article has a big impact on the results.

### Association between metabolic syndrome and hepatocellular carcinoma

To explore potential sources of heterogeneity, we also performed subgroup analysis for different liver events, such as hepatocellular carcinoma, liver-related death, and cirrhosis.

**Fig. 4:**
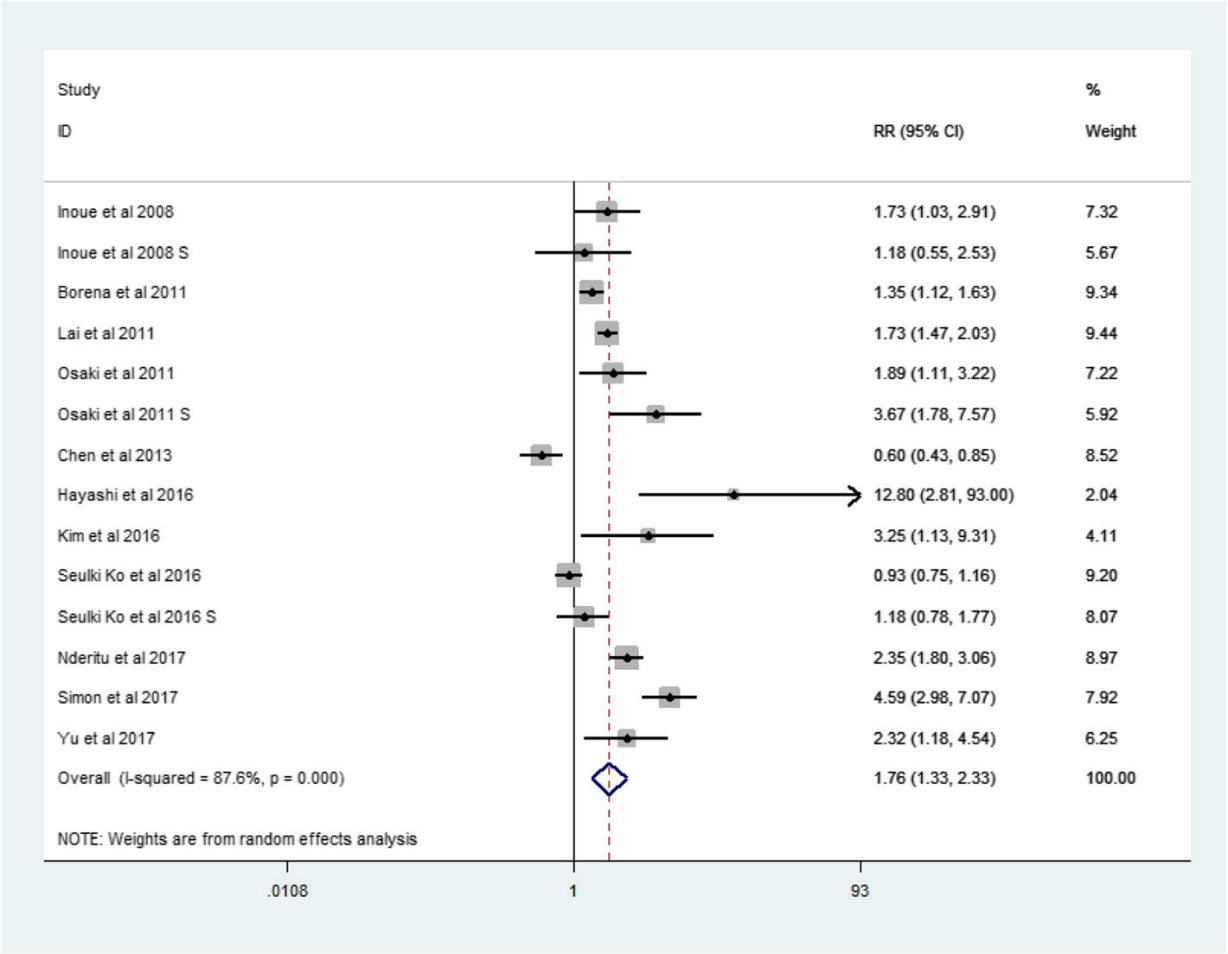
Forest plot: Meta-analysis of the association between metabolic syndrome and HCC

There were 11 studies about hepatocellular carcinoma, which were cohort studies (Table 1). The RR for hepatocellular carcinoma was 1.76 (95% CI: 1.33–2.33, *p* = 0.000, I^2^ = 87.6%) (Fig. 4).

### Association between metabolic syndrome and liver-specific death

**Fig. 5:**
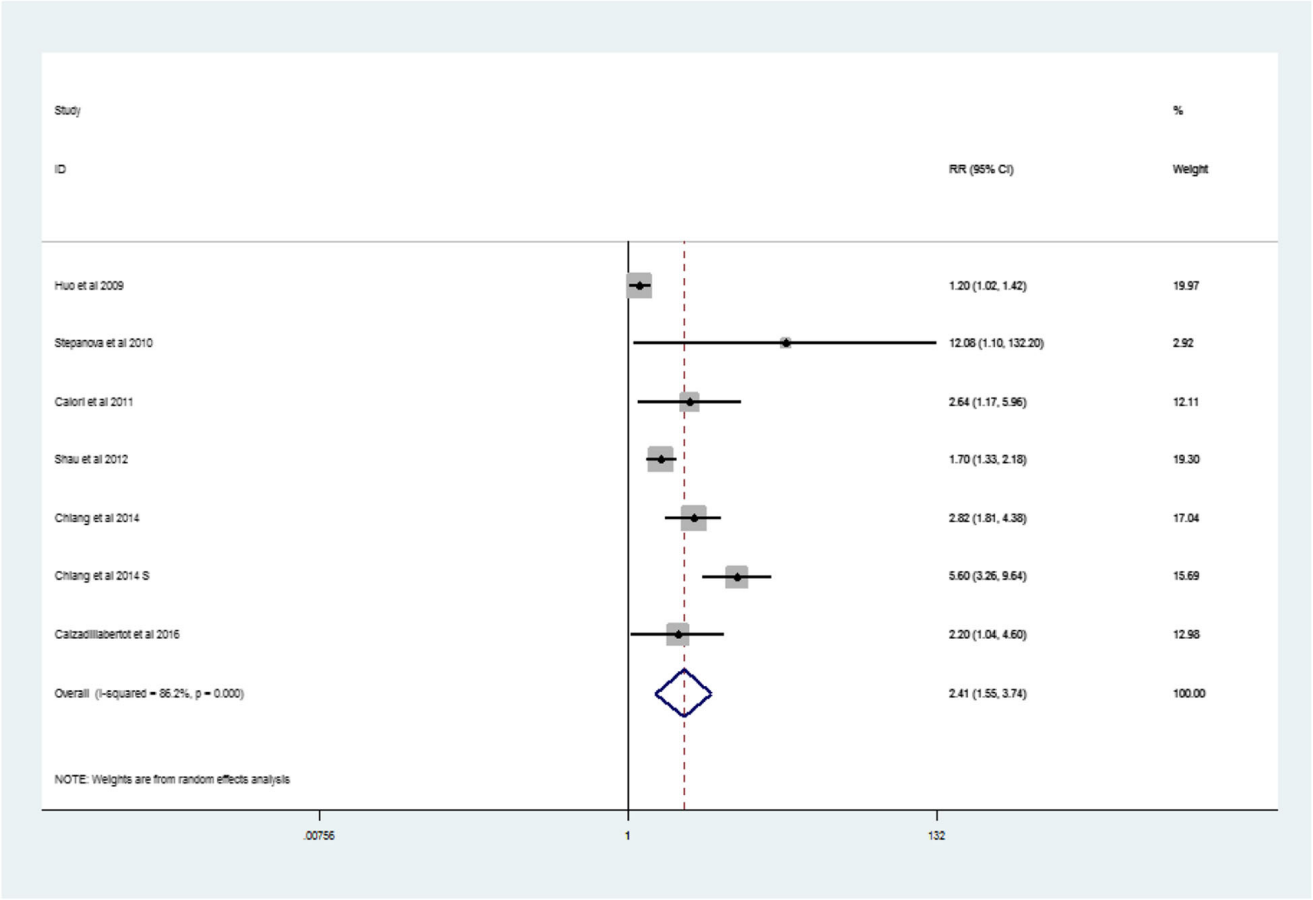
Forest plot: Meta-analysis of the association between metabolic syndrome and liver-related deaths

One case-control study and five cohort studies reported risk estimates for liver-specific death (Table 1). The pooled RR for liver-specific deaths was 2.41 (95% CI: 1.55–3.74, *p* = 0.0005, I^2^ = 86.2%) (Fig. 5).

### Inclusion characteristics

We divided the included population into HBV-positive and HBV-negative groups, and then calculated the relationship between MetS and LREs in each group.

**Fig. 6:**
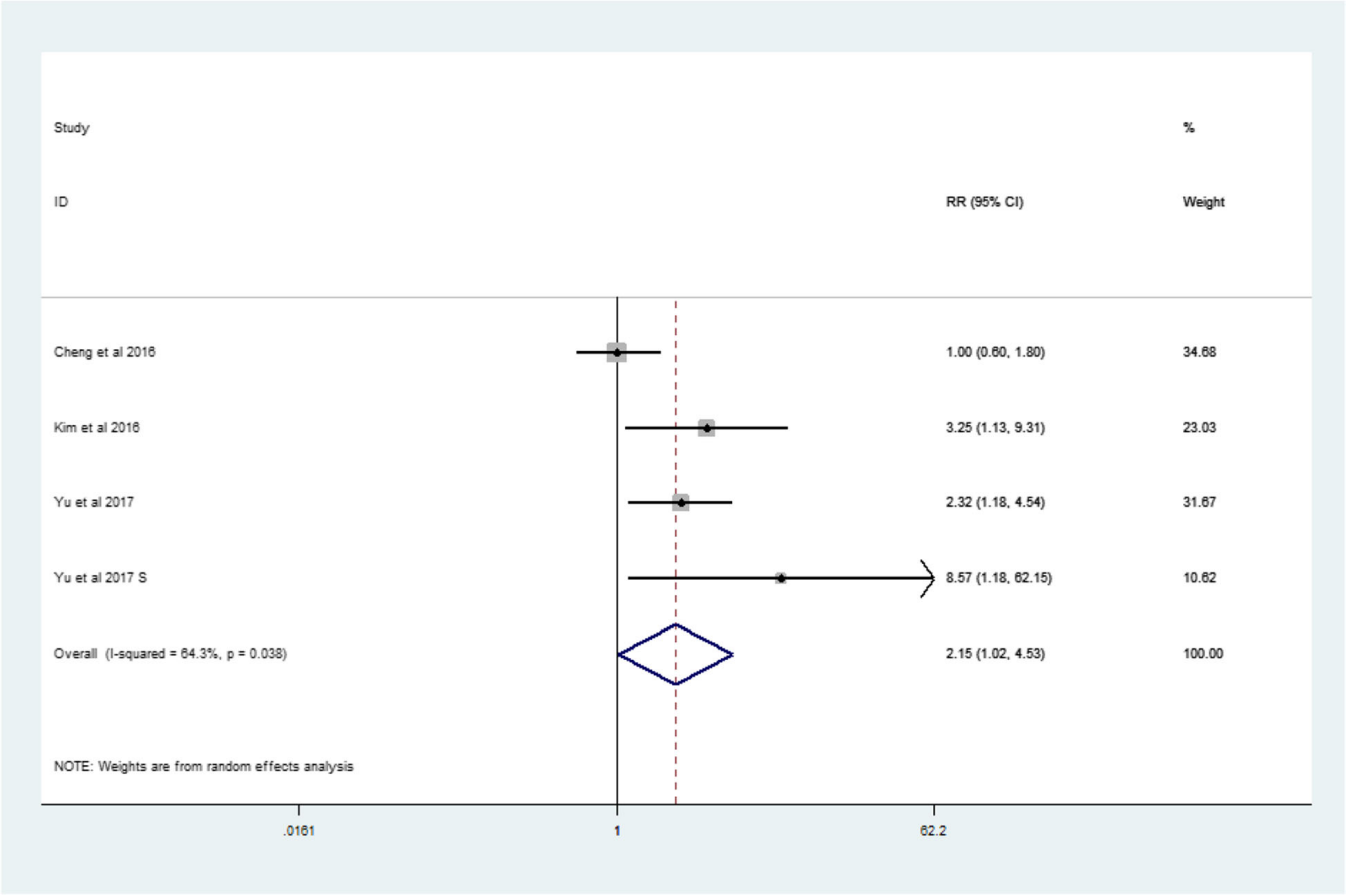
Forest plot: For HBV-positive cases, the association between metabolic syndrome and liver-related events

**Fig. 7:**
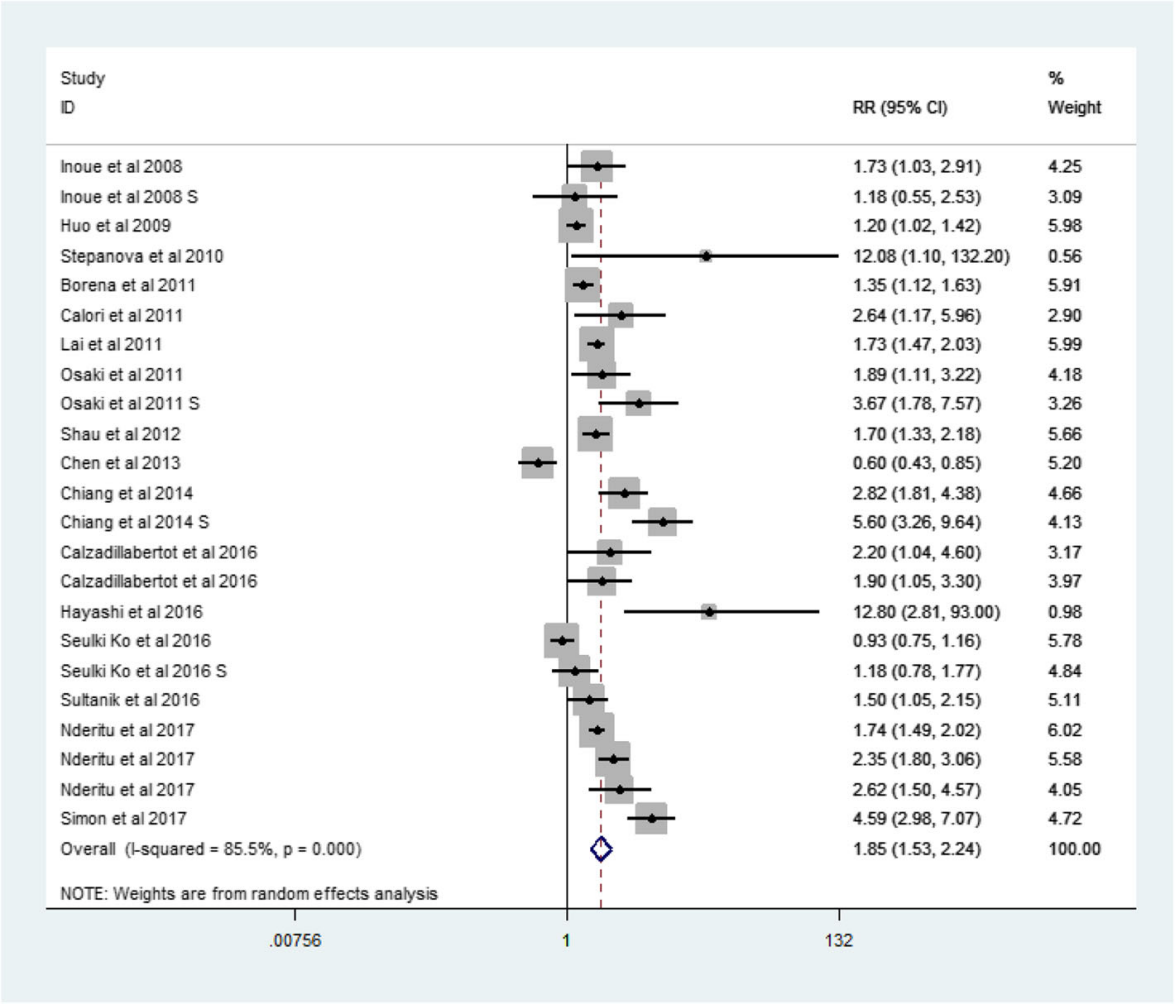
Forest plot: For HBV-negative cases, the association between metabolic syndrome and liver-related events

In the HBV-positive group, the RR for MetS and LREs was 2.15 (95% CI: 1.02–4.53, *p* = 0.038, I2 = 64.3%) (Fig. 6). And in the HBV-negative group, the RR was 1.85 (95% CI: 1.53–2.24, *p* = 0.000, I2 = 85.5%)(Fig. 7).

### Terrain analysis

We divided all the data into Asian and non-Asian regions, and then calculated the relationship between MetS and LREs in each group. We then compared the differences between the groups.

**Fig. 8:**
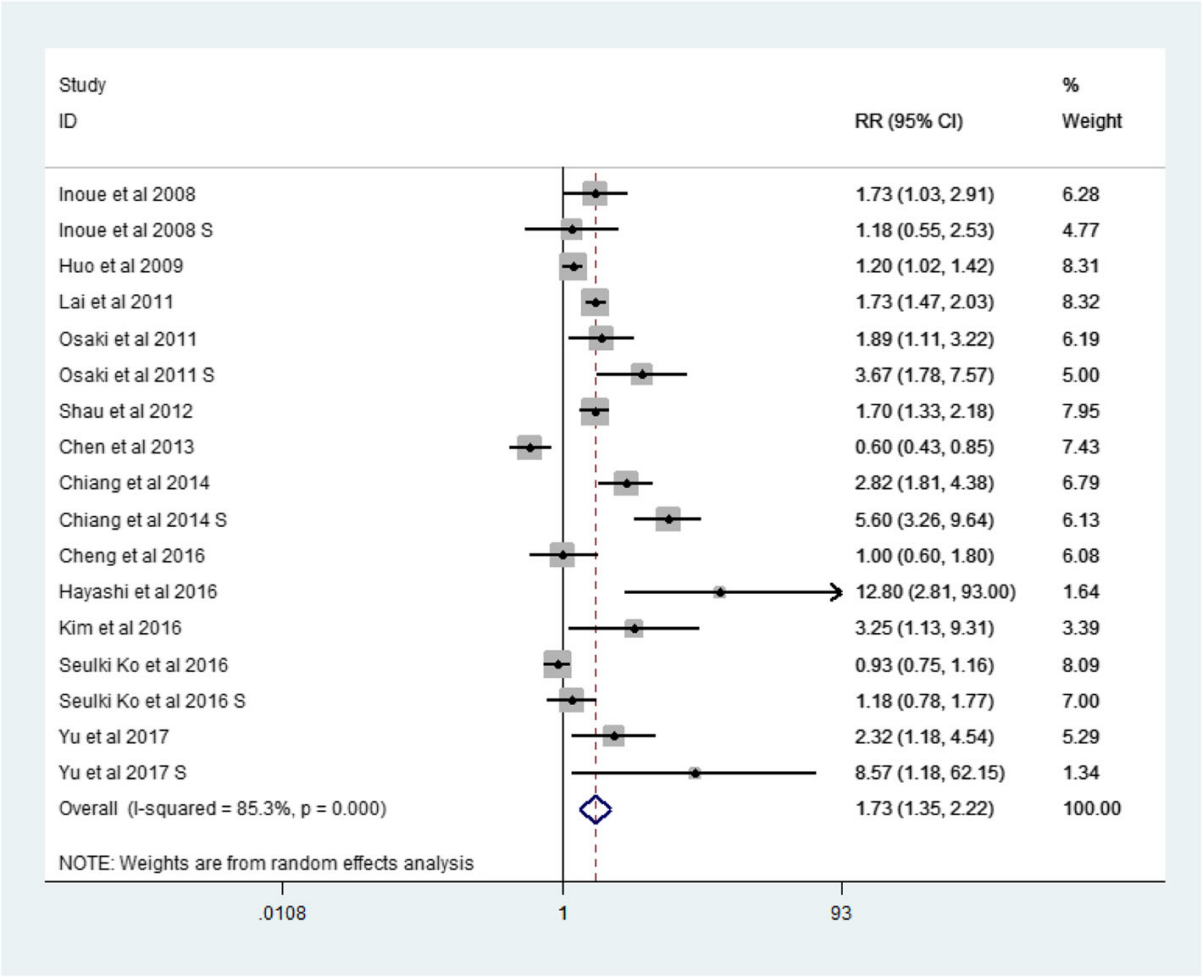
Forest plot: For Asian, the association between metabolic syndrome and liver-related events

**Fig. 9:**
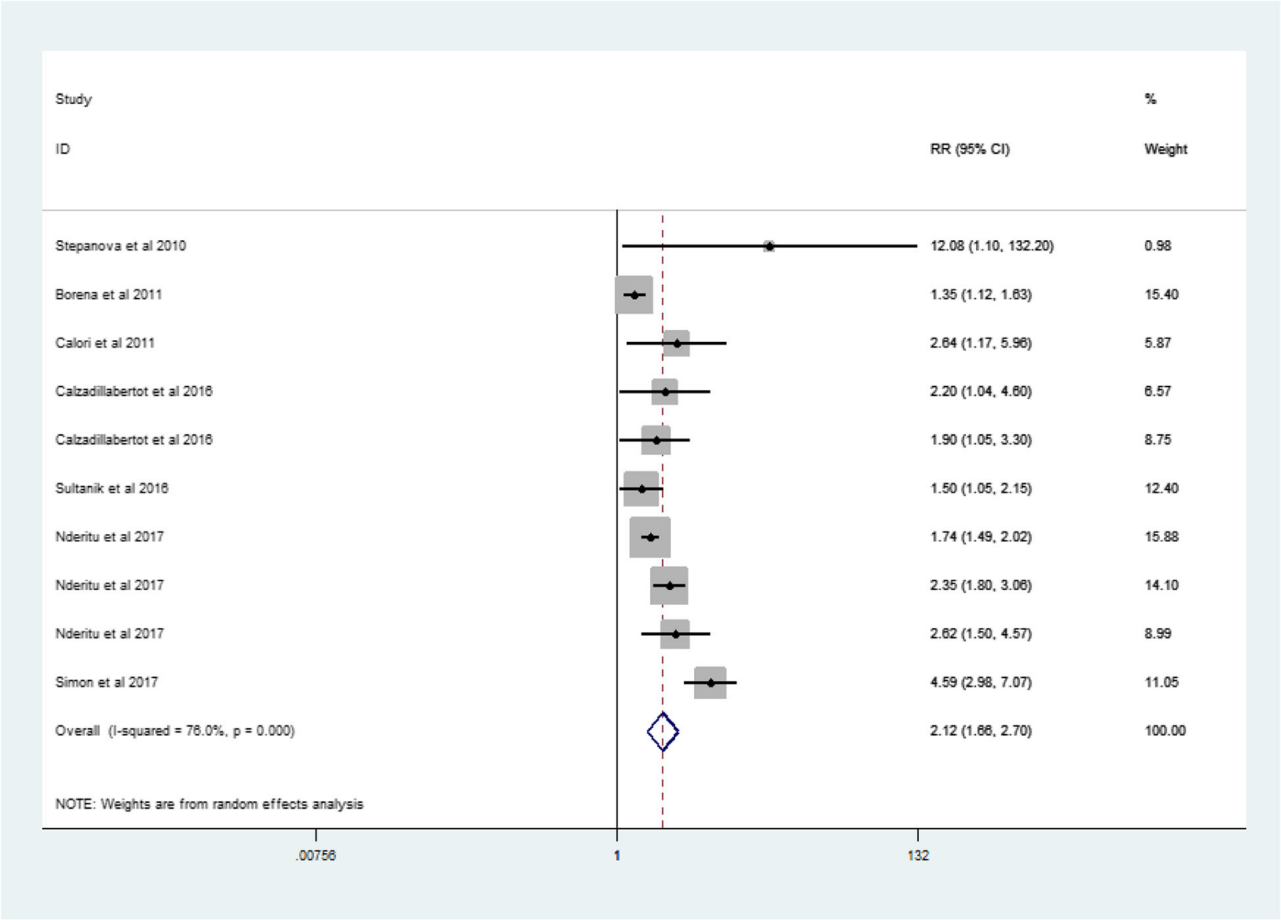
Forest plot: For non-Asian, the association between metabolic syndrome and liver-related events

There were 12 articles from Asian countries (Table 1). The RR for MetS and LREs was 1.73 (95% CI: 1.35–2,22, p = 0.000, I2 = 85.3%)(Fig. 8). Then there were 7 studies describing non-Asian populations (Table 1). The RR for MetS and LREs was 2.12(95% CI: 1.66–2.70, *p* = 0.000, I2 = 76.0%) (Fig. 9) .

### Publication bias

**Fig. 10:**
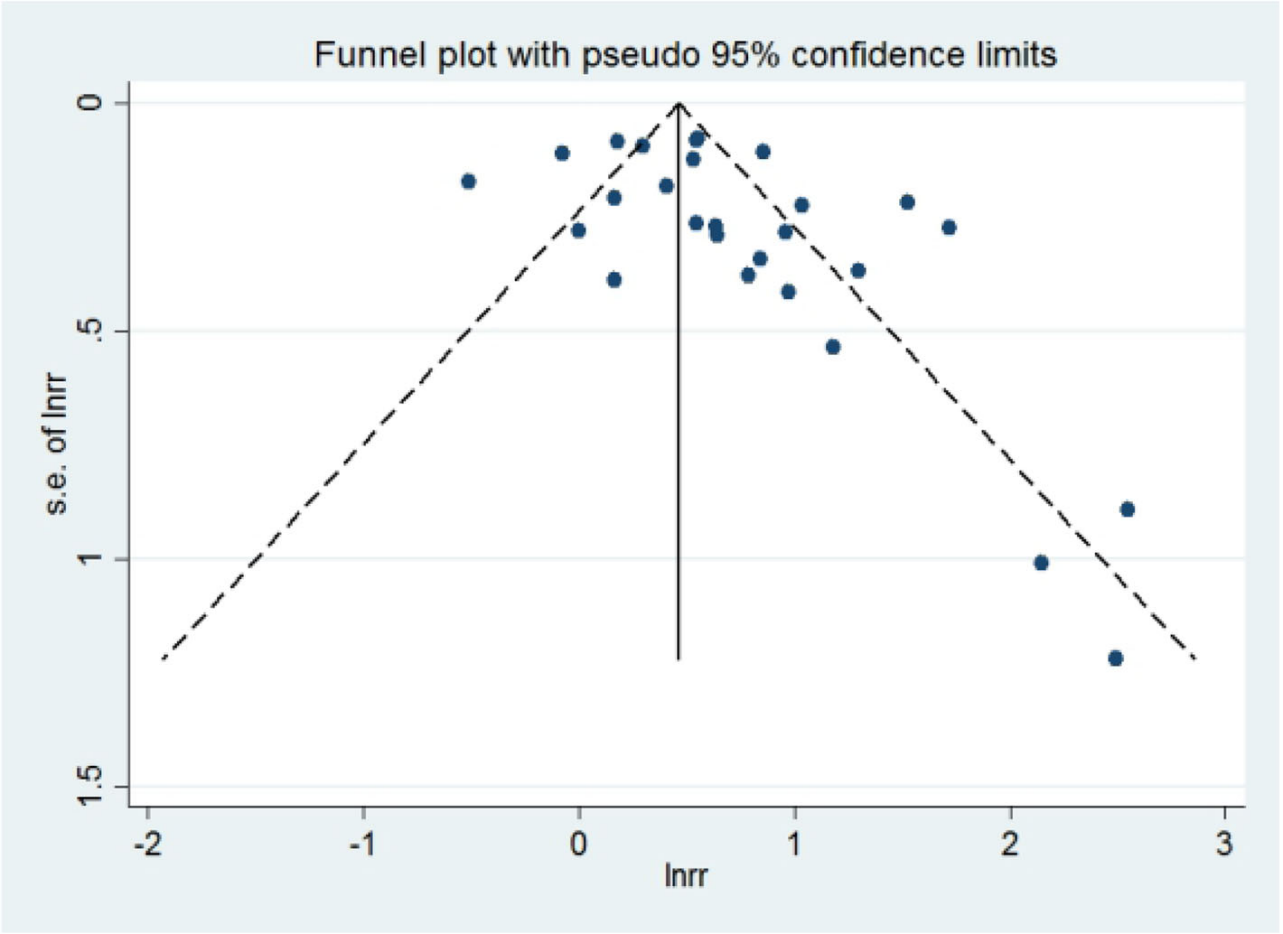
Funnel plots for publication bias

We used the NOS to assess publication quality and determined credible results with 8.2 stars for the 18 cohort studies and 8 stars for the single case-control study (Table 1). Funnel plots were made (Figs. 10) and Egger’s test was performed to assess the publication bias in the meta-analysis. The funnel plot was not completely symmetrical, and the *p* values of Egger’s test were close to 0.05 (Begg and Mazumdar’s test: *p* = 0.08; Egger’s test: *p* = 0.049). Thus, the results suggested that publication bias was present in this meta analysis. Then we use the trim and fill method to correct the funnel asymmetry caused by publication bias, and we get a new RR for MetS and LREs which is 1.49(95% CI: 1.40–1.58, *p* = 0.000), and there was no obvious difference compared with our previous results.

## Discussion

In the meta-analysis of 19 studies including 1,561,457 patients with MetS, 16 studies found a statistically significant positive association, while 3 studies showed a negative association. After we removed the negative study indicated by the random-effects model to have affected the results, we also found that MetS significantly increased the risk of LREs. Therefore, the negative study did not change the finding.

The funnel plot was asymmetrical, and the *p* values of Egger’s test showed a publication bias in this meta analysis. However, through the trim and fill method, we obtained a new RR for LREs with MetS of 1.49 (95% CI: 1.40–1.58, p = 0.000), and there was no obvious difference compared with our previous results. Therefore, we concluded that the results were robust, and MetS is associated with a moderately increased risk of LRE prevalence.

The results also indicated that preexisting MetS confers a statistically significant 1.56- or 2.23-fold increased risk for LREs that is independent of other risk factors, an association also observed for its components. MetS can promote the occurrence of cardiovascular diseases, coronary heart disease and other diseases, and the degree of association between MetS and liver cancer is similar to the former [26, 27]. Nonalcoholic fatty liver disease (NAFLD), including non-alcoholic steatohepatitis (NASH), insulin resistance, and several metabolic abnormalities are closely related, thus suggesting a link between metabolic factors and cancer of the liver.

The findings from our study suggested a 1.33- or 2.33-fold increased risk of HCC development in patients with MetS. In addition, the study also demonstrated a 1.55- or 3.74-fold increased risk of liver-related death in patients with MetS. Our results together indicate that MetS is associated with a moderately increased risk of LRE.

Analysis of related factors showed an overall 115% increase in risk of LREs in HBV-positive cases (RR: 2.15, 95% CI: 1.02–4.53). For HBV-negative cases, the probability indicated a 85% increase in risk (RR: 1.85, 95% CI: 1.53–2.24). This conclusion is consistent with viral hepatitis having been identified as a major risk factors for LREs [2, 3], because over time, patients infected with viral hepatitis develop cirrhosis and, eventually, HCC.

And there was some article showed the pooled RR for HCV subjects with IR was 1.89 (95% CI, 1.54–2.33). It is because the presence of the HCV core protein will increase the level of tumor necrosis factor-α, further leading to proteasomal degradation of the insulin receptor substrate, ultimately altering insulin function and promoting the development of IR. At the same time, IR further causes lipid accumulation in the liver and production of reactive oxygen species, and indirectly activates stellate cells, eventually leading to the occurrence of liver fibrosis [28].

The related factor analysis also indicated that non-Asian MetS populations are more likely than Asian populations to have LREs. There was a 73% increase in risk of LREs for Asians and an 112% increase for non-Asians. The reason for this result is not yet clear, and more research is needed to provide an explanation. It is possible that the economy and the typical non-Western diet in Asia may explain the lower risk of MetS. And for Asian, the HBV vaccine is being recommended widespread. Meanwhile, the incidence of T2DM is lower in the Asian population, and it may have influenced the results. Some studies have reported that up to 90% of obese people in Europe have some degree of fatty degeneration in their liver and overweight increases the risk of HCC. The observed association between excess body weight and the increased risk of liver disease seems to support the reports that liver disease in obese individuals may be mediated through the development of NAFLD and NASH [12].

Our study suggests a relationship between MetS and LREs, but the mechanism that links the two is not fully understood. MetS is likely to be a proxy for other cancer risk factors, such as low physical activity, intake of high caloric food producing a high quantity of heat, high dietary fat intake, low fiber intake, and oxidative stress [29].The mechanisms by which DM induces HCC are related to insulin-like growth factor I (IGF-I) or insulin-like growth factor-binding protein-3 (IGFBP-3) [6]. The presence of insulin resistance and hepatic steatosis may support the hypothesis that diabetes promotes HCC, while the growth of insulin-like growth factor-1, the increase in leptin, the decrease in adiponectin and the imbalance of pro-inflammatory/anti-inflammatory cytokines are supported the appeal opinion [30]. There are several articles that study oxidative stress, cytokine effects, and other factors that contribute to the development of NAFLD. And because of the interaction between many metabolic factors, the study of their contribution to liver disease and liver cancer is more complicated. For example, deposition of free fatty acids and their metabolites in liver tissue is associated with hyperinsulinemia and insulin resistance, and they further promote hepatic steatosis [26].

Moreover, diabetic people have a higher risk of HCV infection, and diabetes is also associated with hyperinsulinemia. Both MetS and LREs are a problem worldwide, and growing evidence shows a relationship between MetS and an increased risk of LREs [31]. Insulin resistance and obesity are often thought to be linked to cancer, and diabetes is an independent prognostic factor for several common human malignancies, such as breast, colorectal, and prostate cancer [32, 33]. Therefore, a link between MetS and cancer is also possible. Previous studies have shown that coexisting MetS in Chronic hepatitis B (CHB) patients increases the risk of different kinds of liver complications, such as liver fibrosis progression and liver cirrhosis [34]. The role of diabetes in liver cancer has been extensively studied, and a variety of biological mechanisms support an association [35–38]. For example, diabetes can affect the recurrence of liver cancer through hyperinsulinemia, hyperglycemia, or chronic inflammation.

A previous meta-analysis has described a relationship between hypertension and cancer, thus suggesting that hypertension is associated with an increased risk of cancer death [26]. That study found that people with an average blood pressure of up to one in five have a cancer risk 2.8 times more than those with the lowest blood pressure. Another meta-analysis has examined the relationship between liver cancer and diabetes. Most studies have shown a significant increase in risk, with a combined risk ratio of 2.5 (95% CI, 1.9–3.2) [39]. The above studies show that hypertension and diabetes as metabolic risk factors are significantly associated with the risk of liver disease, and decreasing the risk of MetS may also contribute to the long-term reduction of liver disease.

There are several limitations that should be considered when interpreting the findings of our meta-analysis. First, there are multiple factors that were not considered in the combined RR analysis, such as dietary habits, HCC family history, and other genetic risk factors, owing to the unavailability of these variables in the original study. Second, for diabetes and liver cancer risk meta-analysis, we referred data from observational studies, while those studies may have lower heterogeneity. Third, because chronic liver disease can also cause diabetes, the link between diabetes and HCC may not be exact. Fourth, different controls on confounding factors also influenced the results. Finally, the meta-analysis was limited to English-language studies, which might have introduced publication bias.

However, our study has several strengths; for example, this article included more data than previous studies and examined links between MetS and cirrhosis, as well as liver-related deaths, among other factors. We also analyzed the geographic region and the population for hepatitis B and other parameters. The all cohort studies in our meta-analysis are of high quality and were able to detect potential associations and eliminate the possibility of recall and selection bias.

## Conclusion

In conclusion, this population-based study showed that MetS is an important risk factor for the development of liver disease. Therefore, the control of the globally prevalent MetS may help reduce liver disease. At the same time, the metabolic syndrome and related factors are introduced in the paper. Trying to avoid these risk factors is a better treatment. More well-designed randomized controlled trials or prospective cohort or retrospective cohort studies are urgently needed to improve understanding of this risk.

## Abbreviations

CHB: Chronic hepatitis B
CI: Confidence interval
DM: Diabetes mellitus
HBV: Hepatitis B virus
HCC: Hepatocellular carcinoma
HCV: Hepatitis C virus
HR: Hazard ratio
IR: Insulin resistance
LREs: Liver-related events
MetS: Metabolic syndrome
NAFLD: Non-alcoholic fatty liver disease
NASH: Non-alcoholic steatohepatitis
NOS: Newcastle-Ottawa Scale
RR: Relative risk
